# No causal effects of serum urate levels on the risk of chronic kidney disease: A Mendelian randomization study

**DOI:** 10.1371/journal.pmed.1002725

**Published:** 2019-01-15

**Authors:** Daniel M. Jordan, Hyon K. Choi, Marie Verbanck, Ruth Topless, Hong-Hee Won, Girish Nadkarni, Tony R. Merriman, Ron Do

**Affiliations:** 1 Department of Genetics and Genomic Sciences, Icahn School of Medicine at Mount Sinai, New York, New York, United States of America; 2 Charles Bronfman Institute for Personalized Medicine, Icahn School of Medicine at Mount Sinai, New York, New York, United States of America; 3 Division of Rheumatology, Allergy and Immunology, Massachusetts General Hospital, Harvard Medical School, Boston, Massachusetts, United States of America; 4 Department of Biochemistry, University of Otago, Dunedin, New Zealand; 5 Samsung Advanced Institute for Health Sciences and Technology, Sungkyunkwan University, Seoul, South Korea; 6 Department of Nephrology, Icahn School of Medicine at Mount Sinai, New York, New York, United States of America; Imperial College London, UNITED KINGDOM

## Abstract

**Background:**

Studies have shown strong positive associations between serum urate (SU) levels and chronic kidney disease (CKD) risk; however, whether the relation is causal remains uncertain. We evaluate whether genetic data are consistent with a causal impact of SU level on the risk of CKD and estimated glomerular filtration rate (eGFR).

**Methods and findings:**

We used Mendelian randomization (MR) methods to evaluate the presence of a causal effect. We used aggregated genome-wide association data (*N* = 110,347 for SU, *N* = 69,374 for gout, *N* = 133,413 for eGFR, *N* = 117,165 for CKD), electronic-medical-record-linked UK Biobank data (*N* = 335,212), and population-based cohorts (*N* = 13,425), all in individuals of European ancestry, for SU levels and CKD. Our MR analysis showed that SU has a causal effect on neither eGFR level nor CKD risk across all MR analyses (all *P* > 0.05). These null associations contrasted with our epidemiological association findings from the 4 population-based cohorts (change in eGFR level per 1-mg/dl [59.48 μmol/l] increase in SU: −1.99 ml/min/1.73 m^2^; 95% CI −2.86 to −1.11; *P* = 8.08 × 10^−6^; odds ratio [OR] for CKD: 1.48; 95% CI 1.32 to 1.65; *P* = 1.52 × 10^−11^). In contrast, the same MR approaches showed that SU has a causal effect on the risk of gout (OR estimates ranging from 3.41 to 6.04 per 1-mg/dl increase in SU, all *P* < 10^−3^), which served as a positive control of our approach. Overall, our MR analysis had >99% power to detect a causal effect of SU level on the risk of CKD of the same magnitude as the observed epidemiological association between SU and CKD. Limitations of this study include the lifelong effect of a genetic perturbation not being the same as an acute perturbation, the inability to study non-European populations, and some sample overlap between the datasets used in the study.

**Conclusions:**

Evidence from our series of causal inference approaches using genetics does not support a causal effect of SU level on eGFR level or CKD risk. Reducing SU levels is unlikely to reduce the risk of CKD development.

## Author summary

**Why was this study done?:**

- Epidemiological studies have shown strong correlations between serum urate (SU) levels and chronic kidney disease (CKD) risk.
- Elevated SU levels are often found in patients with CKD, but it is not clear whether high serum urate is a cause of kidney disease or just a common co-occurrence.
- Previous studies examining whether SU levels had a causal effect on CKD were limited due to not having large enough samples to detect a true causal relationship if it existed and/or had limitations related to the methodology.
- Several clinical trials have been started that aim to use urate-lowering medication to prevent CKD.

**What did the authors do and find?:**

- To determine whether SU level has a causal effect on CKD, we used a methodology known as Mendelian randomization to test whether genetic variants known to increase SU level also increased the risk of CKD.
- We used multiple datasets to perform Mendelian randomization analyses, which included meta-analyses performed across multiple population-based cohorts, 4 individual population-based cohorts, and the large electronic-medical-record-linked UK Biobank.
- Across all datasets, we found no significant causal connection between SU level and risk of CKD.

**What do these findings mean?:**

- Our findings do not support a causal role of SU level in CKD.
- Lower SU levels would be unlikely to translate into reduced risk of CKD.

## Introduction

Approximately 10% of the global population has chronic kidney disease (CKD) [1,2], which can result in end-stage renal disease, associated with shortened life expectancy and requirement for dialysis or kidney transplantation [3]. There are limited therapeutic options for CKD, with management predominantly focused on control of blood pressure, diabetes, and complications. Hence, there is an intense search for novel therapeutic targets.

Observational studies have consistently shown strong positive associations between serum urate (SU) levels and the risk of CKD [4,5]; however, whether the relation is causal remains unknown. Speculated mechanisms for the potential impact of SU levels on CKD have included nitric oxide and renin-angiotensin pathways [6], stimulation of the renin-angiotensin system [7], and vascular smooth muscle cell proliferation [8]. Furthermore, findings in induced-hyperuricemia rodent models have suggested a causal role of urate in hypertension and associated renal pathophysiology [9]. However, these findings are difficult to directly translate to humans as rodents have considerably lower urate levels owing to functional uricase [9].

As effective medications to lower SU levels are available, the potential causal role of SU level in CKD has become one of the most investigated targets for a renoprotective agent. As such, clinical trials of xanthine oxidase inhibitors (allopurinol or febuxostat) for the endpoint of CKD progression/development are currently underway [10].

Genetic epidemiology can be used to evaluate the causality of risk factors with respect to potential endpoints of interest. This approach, called Mendelian randomization (MR), posits that causality can be inferred because the alleles of a particular exposure-associated genotype are assigned randomly at conception. This can minimize the bias that can occur by confounding and reverse causation in conventional observational studies [11]. Genotypes can be used as a genetically determined lifetime exposure of interest and can be tested for a causal effect on outcomes of interest. For example, MR studies have found that low-density lipoprotein cholesterol is causally related to the risk of coronary artery disease, whereas high-density lipoprotein cholesterol is not [12].

Our objective was to evaluate whether genetic data are consistent with a causal effect of SU levels on estimated glomerular filtration rate (eGFR) and risk of CKD. We performed a series of MR analyses of SU level and eGFR and CKD using genetic variants influencing SU level as the exposure without the influence of confounders.

## Methods

### Study design overview

**Fig 1:**
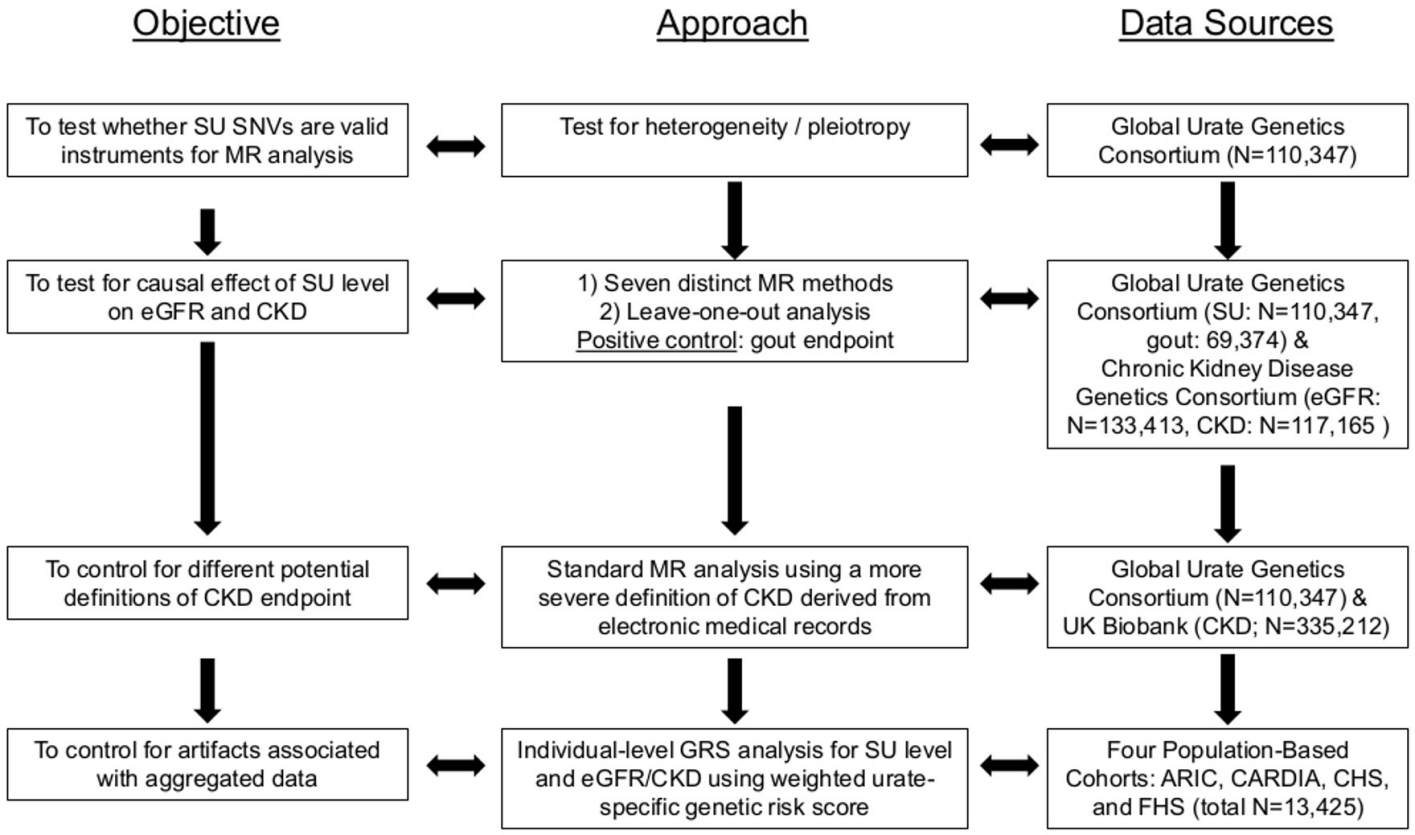
Study design overview. Overview of the study design. ARIC, Atherosclerosis Risk in Communities; CARDIA, Coronary Artery Risk Development in Young Adults; CHS, Cardiovascular Health Study; CKD, chronic kidney disease; eGFR, estimated glomerular filtration rate; FHS, Framingham Heart Study; GRS, genetic risk score; MR, Mendelian randomization; SNV, single nucleotide variant; SU, serum urate.

Our study approach was composed of several complementary components (Fig 1). We first performed a test of heterogeneity to detect the presence of pleiotropy [13] in urate-associated single nucleotide variants (SNVs) identified in a large meta-analysis of genome-wide association (GWA) studies of SU (see S1 Text) [14]. We then conducted 7 distinct MR analyses to evaluate the potential causal role of SU level in eGFR and CKD risk. Since we found significant heterogeneity (see Results), indicating potential pleiotropy, these 7 analytic approaches were specifically chosen to be robust to heterogeneity and pleiotropy in our MR analysis (see S1 Text and below for details). As a positive control endpoint, we assessed whether the same genetic instrumental variables showed the known causal effect of SU on gout. To assess the effect of varying disease endpoint definitions and to control for potential artifacts produced by meta-analyses of heterogeneous populations, we conducted MR analyses using the same SNVs in the UK Biobank, as well as individual-level MR analyses based on 4 population-based cohorts (Atherosclerosis Risk in Communities [ARIC] [15], Coronary Artery Risk Development in Young Adults [CARDIA] [16], Cardiovascular Health Study [CHS] [17], and Framingham Heart Study [FHS] [18]) (Fig 1).

### Data sources and participants

We examined 26 SNVs strongly associated with SU level identified in a GWA meta-analysis study of 110,347 participants of European ancestry conducted by the Global Urate Genetics Consortium (S1 Table) [14].

For MR analyses for eGFR and CKD endpoints, we retrieved GWA study summary statistics for eGFR and CKD from a published meta-analysis conducted by the Chronic Kidney Disease Genetics Consortium (CKDGen) [19]. We used summary statistics for eGFR values calculated from serum creatinine, available in up to 133,413 participants of European ancestry, and for CKD status defined as eGFR < 60 ml/min/1.73 m^2^, available in up to 12,385 cases and 104,780 controls.

For MR analyses for gout (as a positive control endpoint), we retrieved GWA study summary statistics (effect sizes and standard errors) for gout from a published meta-analysis in 2,115 cases and 67,259 controls of European ancestry from the Global Urate Genetics Consortium [14].

Our additional datasets consisted of electronic-medical-record-linked UK Biobank data (*N* = 335,212), and 4 population-based cohorts (ARIC, CARDIA, CHS, and FHS; *N* = 13,425 total). In the UK Biobank, based on recommendations from UK Biobank (http://www.ukbiobank.ac.uk/wp-content/uploads/2018/03/ukb_genetic_data_description_v3.txt), we excluded samples that belonged to any of the following categories: outliers in heterozygosity and missing rates, putative sex chromosome aneuploidy, self-reported non-white British ancestry, and related individuals. Excluded related individuals were defined as 1 individual in each pair with relatedness up to the third degree. In total, 335,212 individuals of white British ancestry remained for analyses. For samples from the UK Biobank, we treated the following ICD-10 codes as indicative of CKD: N03 (chronic nephritic syndrome), N05 (unspecified nephritic syndrome), N17.1 (acute kidney failure with acute cortical necrosis), N17.2 (acute kidney failure with medullary necrosis), N18 (chronic kidney disease), N19 (unspecified kidney failure), N26.9 (renal sclerosis, unspecified), N25.0 (renal osteodystrophy), R80.2 (orthostatic proteinuria, unspecified), I12 (hypertensive chronic kidney disease), I13 (hypertensive heart and chronic kidney disease), Z99.2 (dependence on renal dialysis), Z94.0 (kidney transplant status), and Z49 (encounter for care involving renal dialysis). In total, this produced 5,615 cases. We performed logistic regression for each of the 26 SU-associated SNVs to generate association summary statistics for CKD using this case definition.

In the 4 population-based cohorts (ARIC, CARDIA, CHS, and FHS), we conducted individual-level analyses using a genetic risk score (GRS) of the 26 SNVs associated with SU level. eGFR was recalculated in the 4 prospective cohorts using the Modification of Diet in Renal Disease (MDRD) 4-variable equation. CKD cases were defined as individuals who had an eGFR less than 60 ml/min/1.73 m². A description of the 4 cohorts is provided in the S2 Text.

### Statistical analyses

First, we tested our 26 instrumental SNVs for heterogeneity/pleiotropy using a published test for heterogeneity, the MR-PRESSO [13] global test (see S1 Text for details). We noted the presence of significant heterogeneity/pleiotropy. Accordingly, we selected 7 MR analyses designed to be robust to the presence of heterogeneity in instrumental variables: (i) inverse variance weighted least squares (WLS) regression with a random effects model [20], (ii) MR-Egger regression [21,22], (iii) and (iv) weighted and unweighted median tests [22], (v) and (vi) weighted and unweighted mode-based estimates, and (vii) WLS regression after removing outliers identified by the MR-PRESSO outlier test [13]. For all methods, we used the effect size for the outcome variable (eGFR or CKD) as the response variable, the effect size for the exposure variable (SU) as the predictor variable, and, for weighted methods, the inverse square of the standard error for the outcome variable (eGFR or CKD) as the weight. We used the same 7 methods for analysis of the UK Biobank data. For details of all these methods, see S1 Text. To test the robustness of our analysis to the choice of SNVs, we conducted a leave-one-out analysis, removing each urate-specific SNV separately from the WLS regression test. We also conducted an analysis specifically excluding 2 SNVs: rs12498742 in the *SLC2A9* gene and rs2231142 in the *ABCG2* gene. These are the 2 SNVs with the most significant effects on SU, and are also the only 2 SNVs identified in the SU GWA analysis as having significant sex-specific effects after applying multiple test correction [14].

For our individual-level analyses in the 4 population-based cohorts (ARIC, CARDIA, FHS, and CHS), we calculated the weighted GRS per individual based on the number of risk alleles for the SNVs and the effect size of these SNVs on SU level based on summary results from the Global Urate Genetics Consortium [14]. Individual-level analyses in these cohorts were performed using linear regression when the outcome was a continuous trait or logistic regression when the outcome was a dichotomous trait, after adjustment for age and sex. An inverse variance weighted meta-analysis was performed across the 4 cohorts. To account for relatedness in the FHS cohort, a linear mixed-effects kinship model implemented in the coxme package [23] was used. To account for relatedness in the ARIC cohort, 66 individuals with known family relationships were removed. The other cohorts are not known to have kinship between individuals. To account for possible nonlinearity in the causal relationship between SU and eGFR, the weighted GRS per individual was stratified into 100 strata to calculate localized average causal effect (LACE), and a fractional polynomial fit was performed [24]. The fractional polynomial fit was performed using the mfp package [25].

Epidemiological associations between SU level and eGFR were assessed using linear regression, and between SU level and CKD using logistic regression, adjusting for age and sex in the 4 population-based cohort studies.

We performed analytic power calculations using mRnd [26] based on the strength of observed epidemiological associations of SU with CKD in the 4 population-based cohorts (odds ratio [OR] = 1.34–1.74 per 1 mg/dl [59.48 μmol/l] SU), the fraction of variance in SU explained by each SNV in the Global Urate Genetics Consortium meta-analysis (0.5% to 3%), and the numbers of cases and controls in the CKDGen meta-analysis (12,385 cases and 104,780 controls).

All statistical analyses were performed using R (R Project for Statistical Computing, Vienna, Austria) or Python 2.7 (Python Software Foundation, Wilmington, DE, US).

## Results

### Primary MR analysis for eGFR level and CKD endpoints

**Fig 2:**
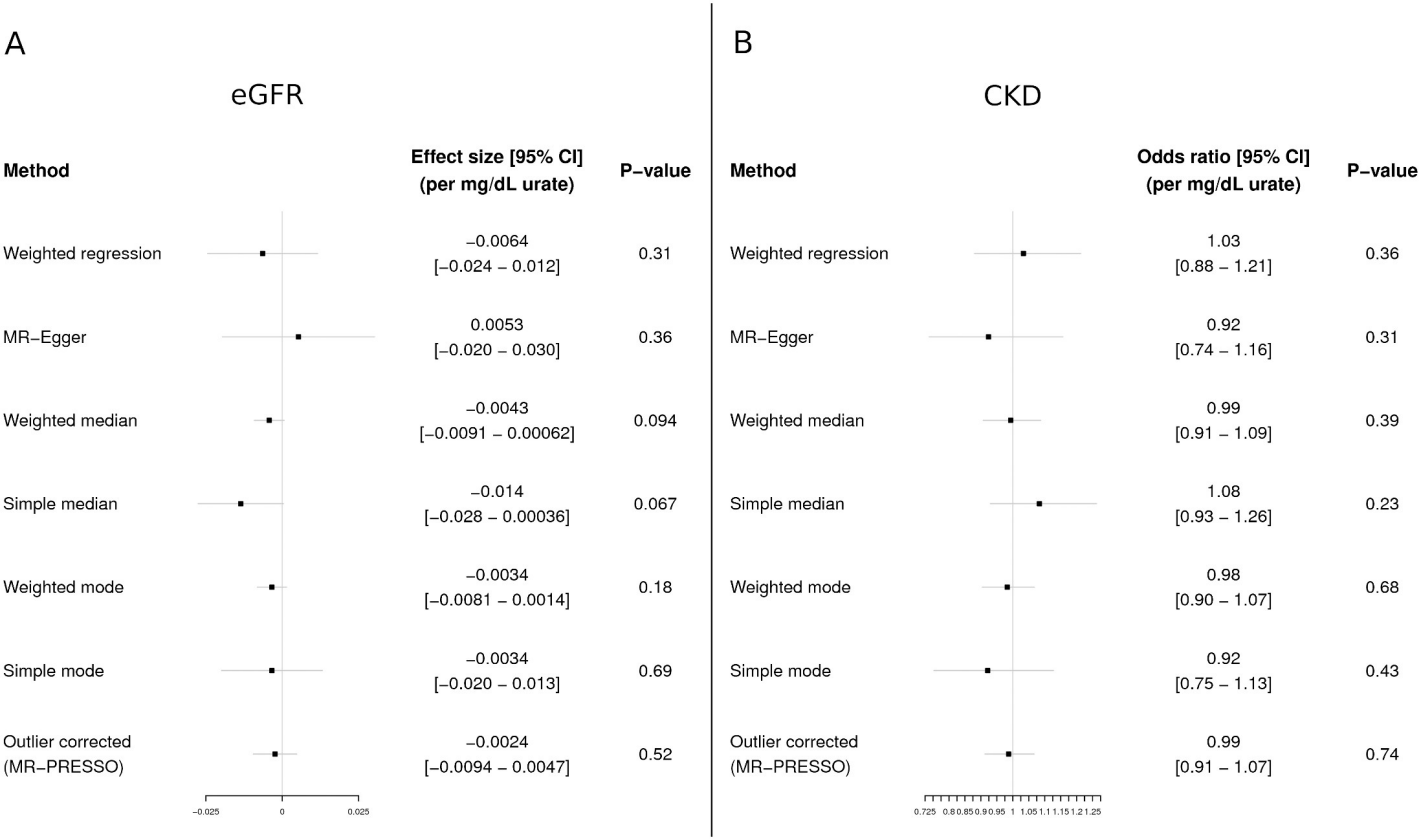
MR analysis of the effect of SU on eGFR and CKD. Estimates of causal effects of serum urate on eGFR (A) and CKD (B) by 7 MR analyses. Effects are shown per 1-mg/dl increase of serum urate. The effect sizes/odds ratios and *P* values were calculated using all single nucleotide variants (SNVs), except for the outlier-corrected MR, where pleiotropic SNVs (detected by MR-PRESSO) were excluded. In the outlier-corrected analysis, 11 outliers were removed for eGFR and 4 outliers were removed for CKD (see S2 Table). CKD, chronic kidney disease; MR, Mendelian randomization; SNV, single nucleotide variant; SU, serum urate.

**Fig 3:**
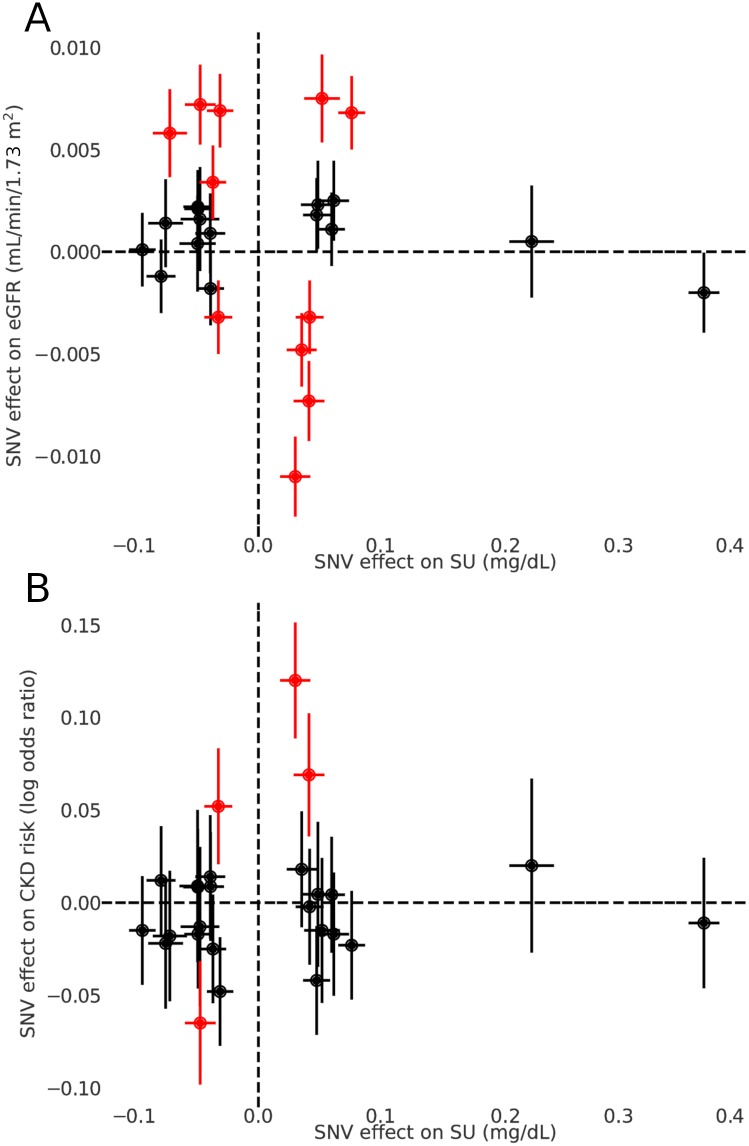
Effects of individual SNVs on SU, eGFR, and CKD. Effects of individual SNVs on SU (*x-*axis, both panels), eGFR (*y-*axis, A), and CKD (*y-*axis, B), as estimated by the respective genome-wide association meta-analyses. Error bars indicate 95% confidence intervals. SNVs identified as outliers by the MR-PRESSO outlier test are highlighted in red. CKD, chronic kidney disease; MR, Mendelian randomization; SNV, single nucleotide variant; SU, serum urate.

We utilized 26 SNVs associated with SU level identified from the GWA study for SU [14] for our MR analyses (S1 Table). We detected significant heterogeneity when testing for a causal effect of SU on both CKD and eGFR (both *P* < 10^−6^ for MR-PRESSO global test), which may indicate the presence of pleiotropy. Accordingly, we selected 7 MR approaches considered to be appropriate for instrumental variables displaying potential pleiotropy: a standard WLS regression analysis with a random effects model, 5 alternative MR methods designed to be robust to heterogeneity, and a WLS regression analysis after removing 4 SNVs identified as outliers for the effect of SU on CKD and 11 SNVs identified as outliers for the effect of SU on eGFR (S2 Table). All 7 approaches detected no causal relationship between SU level and eGFR level (Figs 2A and 3A). Similarly, there was no causal effect detected on CKD risk (Figs 2B and 3B). We observed no heterogeneity/pleiotropy after removal of outliers in the MR test of the effect of SU on CKD (*P* = 0.06); heterogeneity/pleiotropy in the eGFR analysis was still present but reduced (*P* = 0.004). We also performed leave-one-out analysis with the WLS regression method (S1 Fig), as well as an analysis excluding the 2 SNVs with the most significant effects on SU (*SLC2A9* rs12498742 and *ABCG2* rs2231142), which are also known to have sex-specific effects (S2 Fig). Both analyses showed no causal effect.

### MR analysis for gout endpoint

As a positive control, we repeated the same procedure using the same 26 SNVs associated with SU to test for a causal effect of SU on gout. Similarly to the analyses of eGFR and CKD, we observed significant heterogeneity among the 26 SNVs (*P* < 10^−6^ for MR-PRESSO global test). However, unlike for eGFR and CKD, all 7 approaches showed a highly significant causal effect of SU on gout (*P* < 10^−3^ for all tests; S3 Fig). We observed no heterogeneity/pleiotropy after removal of outliers in the MR test of SU’s effect on gout (*P* = 0.09). This result serves as a positive control of our approach as it is consistent with the known causal role of SU in gout [27].

### MR analysis in the UK Biobank

To account for varying definitions of the CKD endpoint, we performed an additional analysis using a clinical definition of kidney disease based on electronic medical records in the UK Biobank. In total, we identified 5,615 CKD cases and 329,597 controls using this definition. We applied the same 7 MR analyses to the 26 SU-associated SNVs. We observed no significant causal relationship of SU level with kidney disease (*P* > 0.05 for all analyses; S4 Fig).

### Individual-level analysis in 4 population-based cohorts

To account for limitations of using aggregated GWA data, such as heterogeneity among study populations in the meta-analyses or unaccounted for population stratification within the sample, we performed an individual-level analysis on 4 population-based cohorts. Within these cohorts, we observed a highly significant association of a urate-specific GRS composed of the 26 urate-associated SNVs with SU (beta = 1.06; 95% CI = 1.00 to 1.13; *P* = 7.06 × 10^−211^). However, the GRS was not associated with either eGFR (beta = −0.42; 95% CI = −1.05 to 0.20; *P* = 0.18) or CKD risk (OR = 1.05; 95% CI = 0.89 to 1.23; *P* = 0.59) (Table 1). We repeated the same analysis after stratifying by sex and age, and found similar results (S3 Table). We also tested for a nonlinear causal relationship using a fractional polynomial fit [24]. The nonlinear model does not appear to fit the data better than the linear model (*P* = 0.49), and still produces a null result for a causal effect of SU on CKD (*P* = 0.34).

**Table 1:** Association of a genetic risk score of serum urate single nucleotide variants with serum urate level, eGFR, and CKD in 4 population-based cohorts.

Group	Serum urate level (mg/dl)			eGFR (ml/min/1.73 m^2^)			CKD		
	Beta	95% CI	*P* value	Beta	95% CI	*P* value	OR	95% CI	*P* value
ARIC	1.08	0.98 to 1.19	1.86 × 10^−91^	−0.40	−1.16 to 0.35	0.30	0.98	0.83 to 1.16	0.82
CARDIA	1.05	0.87 to 1.24	7.28 × 10^−29^	−1.16	−3.09 to 0.77	0.24	1.62	0.78 to 3.46	0.20
CHS	1.10	0.93 to 1.26	5.35 × 10^−38^	−0.47	−2.18 to 1.25	0.59	1.14	0.85 to 1.52	0.38
FHS	1.01	0.88 to 1.14	4.82 × 10^−53^	0.33	−1.76 to 2.41	0.76	N/A	N/A	N/A
Meta-analysis	1.06	1.00 to 1.13	7.06 × 10^−211^	−0.42	−1.05 to 0.20	0.18	1.05	0.89 to 1.23	0.59

ARIC, Atherosclerosis Risk in Communities; CARDIA, Coronary Artery Risk Development in Young Adults; CHS, Cardiovascular Health Study; CKD, chronic kidney disease; eGFR, estimated glomerular filtration rate; FHS, Framingham Heart Study; N/A, not applicable; OR, odds ratio.

### Association of SNV in the *SLC2A9* gene with SU, eGFR, and CKD

We also specifically examined rs12498742 in the *SLC2A9* gene, which encodes the GLUT9 transporter (for glucose and urate) in the renal proximal tubule and is the largest contributor to genetic control of SU level, explaining 3% of variance [14]. This SNV was strongly associated with SU (beta = 0.37, *P* < 10^−700^) but neither with eGFR (beta = 0.002, *P* = 0.06) nor with CKD (OR = 0.99, *P* = 0.53).

### Conventional epidemiological association of SU, eGFR, and CKD

These null associations contrasted with conventional epidemiological association analyses. In a meta-analysis of the same 4 population-based cohorts (ARIC, CARDIA, CHS, and FHS), we observed that an equivalent (1 mg/dl) increase in SU level was associated with reduced eGFR level (beta = −1.99; 95% CI −2.86 to −1.11; *P* = 8.08 × 10^−6^) and increased risk of CKD (OR = 1.48; 95% CI 1.32 to 1.65; *P* = 1.52 × 10^−11^), after adjustment for age and sex (Table 2).

**Table 2:** Observational association between serum urate level and eGFR and risk of CKD in 4 population-based cohorts.

Group	eGFR (ml/min/1.73 m^2^)			CKD		
	Beta	95% CI	*P* value	OR	95% CI	*P* value
ARIC	−1.82	−1.99 to −1.65	3.56 × 10^−94^	1.40	1.34 to 1.46	1.17 × 10^−51^
CARDIA	−1.01	−1.52 to −0.49	1.40 × 10^−4^	1.39	1.14 to 1.68	9.51 × 10^−4^
CHS	−3.31	−3.67 to −2.95	1.75 × 10^−68^	1.61	1.50 to 1.74	1.68 × 10^−37^
FHS	−1.76	−2.32 to −1.21	3.85 × 10^−10^	N/A	N/A	N/A
Meta-analysis	−1.99	−2.86 to −1.11	8.08 × 10^−6^	1.48	1.32 to 1.65	1.52 × 10^−11^

ARIC, Atherosclerosis Risk in Communities; CARDIA, Coronary Artery Risk Development in Young Adults; CHS, Cardiovascular Health Study; CKD, chronic kidney disease; eGFR, estimated glomerular filtration rate; FHS, Framingham Heart Study; OR, odds ratio.

### Statistical power and the probability of missing a causal effect

We assessed the statistical power of our MR study given the sample size and the variance in SU explained by the 26 SNVs we used as instrumental variables. We calculated that our MR analyses would have greater than 99% power to detect a statistically significant effect at an alpha rate of 5%, if causality between SU and CKD were present at the strength indicated by observational epidemiology (OR = 1.5 per 1 mg/dl of SU) (Table 3).

**Table 3:** Power calculations for MR analyses of the effect of SU on CKD.

OR of CKD per 1 mg/dl of SU	Fraction of variance in SU explained by SNV	Power of MR analysis
1.3	0.5%	94%
1.3	3%	>99%
1.5	0.5%	>99%
1.5	3%	>99%
1.7	0.5%	>99%
1.7	3%	>99%

Power calculations performed with mRnd. OR 1.3–1.7 is the 95% confidence interval of the observational epidemiological association between SU and CKD (Table 2); 0.5%–3% is the range of variance explained reported for single SNVs in the SU genome-wide association study.

CKD, chronic kidney disease; MR, Mendelian randomization; OR, odds ratio; SNV, single nucleotide variant; SU, serum urate.

## Discussion

In this study, we investigated a potential causal role for SU level in the development of CKD using a series of complementary MR analyses. Despite previous observational study findings that SU levels were strongly associated with the risk of incident CKD [4,5], our MR analyses found no evidence for a causal role of SU level for eGFR level or incident CKD. In contrast, our positive control MR analysis demonstrated that SU level was causal for the risk of gout, which is consistent with a previous study that showed similar results [28].

Unlike previous studies based on smaller sample sizes, our power calculations show that our study is sufficiently powered to assess a causal relationship between SU level and CKD. One study that examined only eGFR (not CKD) reported that increased SU levels due to SNVs in urate transporter genes were associated with increased eGFR only in men (which is opposite to the expected epidemiological association) [29]. However, this study did not account for pleiotropy, which may explain these unexpected findings. The second MR analysis reported null findings for both eGFR and CKD; however, the study was not sufficiently powered to assess causal relationships for CKD and also did not account for pleiotropy [30]. A third study observed no causal effect of SU levels on renal function in 3,734 Chinese individuals but observed significant effects in some subpopulations: females, individuals under 65 years, individuals with normal eGFR levels, current smokers, and individuals with high fasting glucose levels [31]. This study similarly did not account for pleiotropy and did not directly test for CKD. Importantly, the sample size of the current study is much larger (*N* > 400,000) and therefore has higher power than the previous studies above. Our failure to detect by MR the expected epidemiological association of SU with CKD/eGFR is therefore not due to lack of power. We suggest instead that it is due to our MR analyses being robust to unknown confounders and reverse causation, which can create positive correlations in observational epidemiological studies.

Clinical trial data on lowering SU levels for preventing CKD progression to date have been conflicting [32,33]. Moreover, no randomized controlled trials have been conducted using targeted interventions to lower SU levels for the prevention of incident CKD, although trials evaluating the role of xanthine oxidase inhibitors in disease populations such as patients with early CKD or type 1 diabetes are ongoing. Furthermore, to date, 2 randomized controlled trials among adolescents with hyperuricemic pre-hypertension or stage-1 hypertension found that urate-lowering therapy lowered blood pressure, a strong risk factor for CKD [34,35], whereas a similarly designed trial among adults did not find such a benefit [36]. Nevertheless, these studies did not address the causal role of urate reduction in the prevention of incident CKD in population-based participants, which would be the relevant context of our study. Furthermore, the potential benefit of xanthine oxidase inhibitors could exclusively come from reducing oxidative stress by inhibiting superoxide generation, rather than from SU reduction [37]. Our findings suggest that SU reduction alone would not result in prevention of incident CKD, consistent with a recent study that showed that the initiation of allopurinol in patients with gout was not associated with a change in CKD risk [38]. Finally, our results are relevant to the impact of lowering urate and do not rule out the potential benefit of superoxide reduction resulting from xanthine oxidase inhibition [37].

Potential limitations of this study and, in particular, MR deserve comment. First, MR analysis requires suitable SNVs to act as instrumental variables, and selection of inappropriate or unrepresentative instrumental variables may undermine the validity of the study. Here, we used the SNVs significantly associated with SU in a previously published GWA study. We also performed an additional analysis using a single SNV with a strong effect on SU and a well-understood mechanism (rs12498742 in the *SLC2A9* gene), as well as an analysis excluding both this SNV and a second large-effect SNV (rs2231142 in the *ABCG2* gene). Second, an assumption of MR is that the SNVs used as instrumental variables are not subject to “horizontal pleiotropy,” meaning that the SNVs should not have pleiotropic effects on the outcome outside of the target biomarker or risk factor. We have used MR approaches designed to be robust to horizontal pleiotropy to account for this. Third, our MR findings cannot predict with absolute certainty that therapeutics lowering SU levels will not result in a lowering of CKD risk, since the effects of genetic variation may not be exactly the same as the effects of a therapeutic intervention. Nevertheless, studies have suggested that genetic findings can predict the effect of drugs on disease outcomes [39]. Fourth, the study is focused on individuals of European ancestry; therefore, it is unclear whether our results can be generalized to non-European populations. Fifth, there are some overlapping samples between the SU and CKD GWA datasets (61% of samples in the SU GWA dataset, 50% in the eGFR GWA dataset, and 43% in the CKD GWA dataset), which can affect the accuracy of MR tests [40]. However, this would only affect our study by producing a false positive result, and we observe only negative results. Furthermore, we also performed an MR analysis with non-overlapping datasets (the SU GWA dataset and the UK Biobank dataset for CKD), and found the same negative result. Sixth, the SU and CKD GWA studies both aggregated heterogeneous populations with differing distributions of age, sex, and other potentially important features, and it is possible that the differences between the populations mask the effect of SU on CKD. However, these studies did account for age and sex as covariates, and we performed individual-level analyses that found a negative result when stratifying by age and sex. Seventh, some individuals included in the GWA datasets for SU and CKD may have been taking urate-lowering medication, which may distort the relationship between SU and CKD. We were not able to remove these individuals from our sample for the GWA summary statistic analysis. However, we did remove individuals taking urate-lowering medication from our individual-level analyses in the population-based cohorts, and these analyses found a similar result to the GWA summary statistic analysis.

In conclusion, our MR analyses do not support a causal effect of SU level on eGFR or CKD. Our results suggest that lowering SU levels would be unlikely to translate into risk reduction for incident CKD.

## Supporting information

S1 Fig Leave-one-out analysis of effect of SU on CKD using WLS regression. Lines show 95% confidence intervals; odds ratio estimates are per 1 mg/dl of SU.
(TIFF)

S2 Fig MR analyses leaving out the 2 most significant SNVs. Analyses were performed with 24 SNVs, excluding rs12498742 in the *SLC2A9* gene and rs2231142 in the *ABCG2* gene. Lines show 95% confidence intervals; odds ratio estimates are per 1 mg/dl of SU. Four outliers were removed in the outlier-corrected (MR-PRESSO) analysis (see S2 Table).
(TIFF)

S3 Fig MR analyses showing significant causal effect of urate on gout. Lines show 95% confidence intervals; odds ratio estimates are per 1 mg/dl of SU. One outlier was removed in the outlier-corrected (MR-PRESSO) analysis (see S2 Table).
(TIFF)

S4 Fig MR analysis on UK Biobank data. Lines show 95% confidence intervals; odds ratio estimates are per 1 mg/dl of SU. Four outliers were removed in the outlier-corrected (MR-PRESSO) analysis (see S2 Table).
(TIFF)

S1 Table List of SNVs for SU level. (XLSX)

S2 Table Detection of pleiotropy using the MR-PRESSO (Mendelian randomization pleiotropy REsidual sum and outlier) test on SU and eGFR, CKD, and gout. (XLSX)

S3 Table MR results in population-based cohorts stratified by age and sex. (XLSX)

S1 Text Description of the 7 MR methodologies used in the primary analysis. (DOCX)

S2 Text Description of the 4 population-level cohorts. (DOCX)

## Abbreviations

ARIC: Atherosclerosis Risk in Communities
CARDIA: Coronary Artery Risk Development in Young Adults
CHS: Cardiovascular Health Study
CKD: chronic kidney disease
CKDGen: Chronic Kidney Disease Genetics Consortium
eGFR: estimated glomerular filtration rate
FHS: Framingham Heart Study
GRS: genetic risk score
GWA: genome-wide association
MR: Mendelian randomization
OR: odds ratio
SNV: single nucleotide variant
SU: serum urate
WLS: weighted least squares

## References

[1] Xie Y, Bowe B, Mokdad AH, Xian H, Yan Y, Li T, et al Analysis of the Global Burden of Disease study highlights the global, regional, and national trends of chronic kidney disease epidemiology from 1990 to 2016. Kidney Int. 2018;94(3):567–81. 10.1016/j.kint.2018.04.011 30078514

[2] National Kidney Foundation. Global facts: about kidney disease. New York: National Kidney Foundation; 2017 [cited 2018 Dec 13]. https://www.kidney.org/kidneydisease/global-facts-about-kidney-disease.

[3] Levey AS, Stevens LA, Coresh J. Conceptual model of CKD: applications and implications. Am J Kidney Dis. 2009;53(3 Suppl 3):S4–16. 10.1053/j.ajkd.2008.07.048 19231760

[4] Weiner DE, Tighiouart H, Elsayed EF, Griffith JL, Salem DN, Levey AS. Uric acid and incident kidney disease in the community. J Am Soc Nephrol. 2008;19(6):1204–11. 10.1681/ASN.2007101075 18337481 PMC2396939

[5] Kumagai T, Ota T, Tamura Y, Chang WX, Shibata S, Uchida S. Time to target uric acid to retard CKD progression. Clin Exp Nephrol. 2016;21:182 10.1007/s10157-016-1288-2 27339448

[6] Johnson RJ, Kang DH, Feig D, Kivlighn S, Kanellis J, Watanabe S, et al Is there a pathogenetic role for uric acid in hypertension and cardiovascular and renal disease? Hypertension. 2003;41(6):1183–90. 10.1161/01.HYP.0000069700.62727.C5 12707287

[7] Perlstein TS, Gumieniak O, Hopkins PN, Murphey LJ, Brown NJ, Williams GH, et al Uric acid and the state of the intrarenal renin-angiotensin system in humans. Kidney Int. 2004;66(4):1465–70. 10.1111/j.1523-1755.2004.00909.x 15458439

[8] Rao GN, Corson MA, Berk BC. Uric acid stimulates vascular smooth muscle cell proliferation by increasing platelet-derived growth factor A-chain expression. J Biol Chem. 1991;266(13):8604–8. 2022672

[9] Kang DH, Nakagawa T, Feng L, Watanabe S, Han L, Mazzali M, et al A role for uric acid in the progression of renal disease. J Am Soc Nephrol. 2002;13(12):2888–97. 12444207 10.1097/01.asn.0000034910.58454.fd

[10] Hosoya T, Kimura K, Itoh S, Inaba M, Uchida S, Tomino Y, et al The effect of febuxostat to prevent a further reduction in renal function of patients with hyperuricemia who have never had gout and are complicated by chronic kidney disease stage 3: study protocol for a multicenter randomized controlled study. Trials. 2014;15:26 10.1186/1745-6215-15-26 24433285 PMC3899617

[11] Robinson PC, Choi HK, Do R, Merriman TR. Insight into rheumatological cause and effect through the use of Mendelian randomization. Nat Rev Rheumatol. 2016;12(8):486–96. 10.1038/nrrheum.2016.102 27411906

[12] Voight BF, Peloso GM, Orho-Melander M, Frikke-Schmidt R, Barbalic M, Jensen MK, et al Plasma HDL cholesterol and risk of myocardial infarction: a Mendelian randomisation study. Lancet. 2012;380(9841):572–80. 10.1016/S0140-6736(12)60312-2 22607825 PMC3419820

[13] Verbanck M, Chen CY, Neale B, Do R. Detection of widespread horizontal pleiotropy in causal relationships inferred from Mendelian randomization between complex traits and diseases. Nat Genet. 2018;50(5):693–8. 10.1038/s41588-018-0099-7 29686387 PMC6083837

[14] Kottgen A, Albrecht E, Teumer A, Vitart V, Krumsiek J, Hundertmark C, et al Genome-wide association analyses identify 18 new loci associated with serum urate concentrations. Nat Genet. 2013;45(2):145–54. 10.1038/ng.2500 23263486 PMC3663712

[15] The ARIC investigators. The Atherosclerosis Risk in Communities (ARIC) Study: design and objectives. Am J Epidemiol. 1989;129(4):687–702. 2646917

[16] Friedman GD, Cutter GR, Donahue RP, Hughes GH, Hulley SB, Jacobs DR Jr, et al CARDIA: study design, recruitment, and some characteristics of the examined subjects. J Clin Epidemiol. 1988;41(11):1105–16. 3204420 10.1016/0895-4356(88)90080-7

[17] Fried LP, Borhani NO, Enright P, Furberg CD, Gardin JM, Kronmal RA, et al The Cardiovascular Health Study: design and rationale. Ann Epidemiol. 1991;1(3):263–76. 1669507 10.1016/1047-2797(91)90005-w

[18] Mahmood SS, Levy D, Vasan RS, Wang TJ. The Framingham Heart Study and the epidemiology of cardiovascular disease: a historical perspective. Lancet. 2014;383(9921):999–1008. 10.1016/S0140-6736(13)61752-3 24084292 PMC4159698

[19] Pattaro C, Teumer A, Gorski M, Chu AY, Li M, Mijatovic V, et al Genetic associations at 53 loci highlight cell types and biological pathways relevant for kidney function. Nat Commun. 2016;7:10023 10.1038/ncomms10023 26831199 PMC4735748

[20] Burgess S, Butterworth A, Thompson SG. Mendelian randomization analysis with multiple genetic variants using summarized data. Genet Epidemiol. 2013;37(7):658–65. 10.1002/gepi.21758 24114802 PMC4377079

[21] Bowden J, Davey Smith G, Burgess S. Mendelian randomization with invalid instruments: effect estimation and bias detection through Egger regression. Int J Epidemiol. 2015;44(2):512–25. 10.1093/ije/dyv080 26050253 PMC4469799

[22] Burgess S, Bowden J, Fall T, Ingelsson E, Thompson SG. Sensitivity analyses for robust causal inference from Mendelian randomization analyses with multiple genetic variants. Epidemiology. 2017;28(1):30–42. 10.1097/EDE.0000000000000559 27749700 PMC5133381

[23] Therneau TM. coxme: mixed effects Cox models R package version 2.2–10. Vienna: R Project for Statistical Computing; 2018.

[24] Staley JR, Burgess S. Semiparametric methods for estimation of a nonlinear exposure-outcome relationship using instrumental variables with application to Mendelian randomization. Genet Epidemiol. 2017;41(4):341–52. 10.1002/gepi.22041 28317167 PMC5400068

[25] Ambler G, Benner A. mfp: multivariable fractional polynomials R package version 1.5.2. Vienna: R Project for Statistical Computing; 2015.

[26] Brion M-JA, Shakhbazov K, Visscher PM. Calculating statistical power in Mendelian randomization studies. Int J Epidemiol. 2013;42(5):1497–501. 10.1093/ije/dyt179 24159078 PMC3807619

[27] Choi HK, Mount DB, Reginato AM. Pathogenesis of gout. Ann Intern Med. 2005;143:499–516. 16204163 10.7326/0003-4819-143-7-200510040-00009

[28] Keenan T, Zhao W, Rasheed A, Ho WK, Malik R, Felix JF, et al Causal assessment of serum urate levels in cardiometabolic diseases through a Mendelian randomization study. J Am Coll Cardiol. 2016;67(4):407–16. 10.1016/j.jacc.2015.10.086 26821629 PMC5503188

[29] Hughes K, Flynn T, de Zoysa J, Dalbeth N, Merriman TR. Mendelian randomization analysis associates increased serum urate, due to genetic variation in uric acid transporters, with improved renal function. Kidney Int. 2014;85(2):344–51. 10.1038/ki.2013.353 24048376 PMC5665684

[30] Yang Q, Kottgen A, Dehghan A, Smith AV, Glazer NL, Chen MH, et al Multiple genetic loci influence serum urate levels and their relationship with gout and cardiovascular disease risk factors. Circ Cardiovasc Genet. 2010;3(6):523–30. 10.1161/CIRCGENETICS.109.934455 20884846 PMC3371395

[31] Liu J, Zhang H, Dong Z, Zhou J, Ma Y, Li Y, et al Mendelian randomization analysis indicates serum urate has a causal effect on renal function in Chinese women. Int Urol Nephrol. 2017;49(11):2035–42. 10.1007/s11255-017-1686-8 28856502

[32] Fleeman N, Pilkington G, Dundar Y, Dwan K, Boland A, Dickson R, et al Allopurinol for the treatment of chronic kidney disease: a systematic review. Health Technol Assess. 2014;18(40):1–77, v–vi. 10.3310/hta18400 24965683 PMC4781423

[33] Kanji T, Gandhi M, Clase CM, Yang R. Urate lowering therapy to improve renal outcomes in patients with chronic kidney disease: systematic review and meta-analysis. BMC Nephrol. 2015;16:58 10.1186/s12882-015-0047-z 25928556 PMC4431373

[34] Feig DI, Soletsky B, Johnson RJ. Effect of allopurinol on blood pressure of adolescents with newly diagnosed essential hypertension: a randomized trial. JAMA. 2008;300(8):924–32. 10.1001/jama.300.8.924 18728266 PMC2684336

[35] Soletsky B, Feig DI. Uric acid reduction rectifies prehypertension in obese adolescents. Hypertension. 2012;60(5):1148–56. 10.1161/HYPERTENSIONAHA.112.196980 23006736

[36] McMullan CJ, Borgi L, Fisher N, Curhan G, Forman J. Effect of uric acid lowering on renin-angiotensin-system activation and ambulatory BP: a randomized controlled trial. Clin J Am Soc Nephrol. 2017;12(5):807–16. 10.2215/CJN.10771016 28320765 PMC5477221

[37] George J, Carr E, Davies J, Belch JJ, Struthers A. High-dose allopurinol improves endothelial function by profoundly reducing vascular oxidative stress and not by lowering uric acid. Circulation. 2006;114(23):2508–16. 10.1161/CIRCULATIONAHA.106.651117 17130343

[38] Vargas-Santos AB, Peloquin CE, Zhang Y, Neogi T. Association of chronic kidney disease with allopurinol use in gout treatment. JAMA Int Med. 2018;178(11):1526–33. 10.1001/jamainternmed.2018.4463 30304329 PMC6248199

[39] Nelson MR, Johnson T, Warren L, Hughes AR, Chissoe SL, Xu CF, et al The genetics of drug efficacy: opportunities and challenges. Nat Rev Genet. 2016;17(4):197–206. 10.1038/nrg.2016.12 26972588

[40] Burgess S, Davies NM, Thompson SG. Bias due to participant overlap in two-sample Mendelian randomization. Genet Epidemiol. 2016;40(7):597–608. 10.1002/gepi.21998 27625185 PMC5082560

