# Supplementary material for: No causal effects of serum urate levels on the risk of chronic kidney disease: A Mendelian randomization study
Source: PLoS Med. 2019 Jan 15;16(1):e1002725. doi: 10.1371/journal.pmed.1002725 (PMC6333326; doi:10.1371/journal.pmed.1002725)
Supplement: S1 Text — (DOCX) [file pmed.1002725.s008.docx]

# MR analyses

We conducted seven MR approaches, including i) weighted, random effects, linear regression,[1] ii) weighted, random-effects Egger regression,[2] iii) and iv) weighted and unweighted Median test,[1] v) and vi) weighted and unweighted Mode-Based Estimate,[3] and vii) the same weighted, random effects linear regression after removing horizontal pleiotropic outliers identified by the MR-PRESSO outlier test.[4] For all regression-based tests (i, ii, and vii) we calculated P-values for causal effect using a Wald-style t test. For the median and mode based tests (iii-vi), we calculated P-values using a bootstrap method, as described in the initial publications describing those tests.

For brevity, we use “pleiotropy” to describe horizontal pleiotropy, which occurs when a genetic variant has an effect on outcome outside of the pathway of the exposure. Egger regression is a method that estimates and adjusts for an overall pleiotropic effect on all SNVs. The Median approach and the Mode-Based Estimate are robust to pleiotropic outliers. Therefore, these three methods were performed including all 26 SU SNVs as instrumental variables. In contrast, inverse variance weighted MR methods are more susceptible to pleiotropic outliers. Therefore, as an additional analysis, we accounted for pleiotropy in the inverse variance weighted MR method by applying the MR-PRESSO outlier test, which identifies pleiotropic outlier SNVs that can then be removed from subsequent analyses. We identified outliers using a *P*<0.05 threshold after correcting for multiple testing using the Bonferroni correction. We identified 4 outliers in the analysis of CKD, 11 in the analysis of eGFR, and 1 in the analysis of gout (**S2 Table**).

1. Burgess S, Bowden J, Fall T, Ingelsson E, Thompson SG. Sensitivity Analyses for Robust Causal Inference from Mendelian Randomization Analyses with Multiple Genetic Variants. Epidemiology. 2017;28(1):30-42. doi: 10.1097/EDE.0000000000000559. PubMed PMID: 27749700; PubMed Central PMCID: PMC5133381.

2. Bowden J, Davey Smith G, Burgess S. Mendelian randomization with invalid instruments: effect estimation and bias detection through Egger regression. International journal of epidemiology. 2015;44(2):512-25. Epub 2015/06/08. doi: 10.1093/ije/dyv080. PubMed PMID: 26050253; PubMed Central PMCID: PMC4469799.

3. Hartwig FP, Davey Smith G, Bowden J. Robust inference in summary data Mendelian randomization via the zero modal pleiotropy assumption. Int J Epidemiol. 2017;46(6):1985-98. Epub 2017/10/19. doi: 10.1093/ije/dyx102. PubMed PMID: 29040600; PubMed Central PMCID: PMCPMC5837715.

4. Verbanck M, Chen CY, Neale B, Do R. Detection of widespread horizontal pleiotropy in causal relationships inferred from Mendelian randomization between complex traits and diseases. Nature genetics. 2018;50(5):693-8. doi: 10.1038/s41588-018-0099-7. PubMed PMID: 29686387.
