# Supplementary material for: No causal effects of serum urate levels on the risk of chronic kidney disease: A Mendelian randomization study
Source: PLoS Med. 2019 Jan 15;16(1):e1002725. doi: 10.1371/journal.pmed.1002725 (PMC6333326; doi:10.1371/journal.pmed.1002725)
Supplement: S2 Text — (DOCX) [file pmed.1002725.s009.docx]

# Description of population-based cohorts

Atherosclerosis Risk In Communities (ARIC) was a longitudinal epidemiological study conducted between 1987 and 1998. A total of 15,792 participants were recruited from four communities in the US, and extensive records of demographic, social and medial record made over four exams. 66 samples from ARIC were removed from this analysis due to relatedness.

Coronary Artery Risk Development in Young Adults Study (CARDIA) was designed to study the cardiovascular diseases and associated risk factors. Recruitment began in 1985 with the recruitment of 5115 men and women between ages of 18 and 30. The longitudinal follow-ups ongoing in five year intervals. Exams five and six, which represent collection years ten and fifteen of the CARIDA study respectively, were the only exams where uric acid was measured. Exam six measurements were used if there were no measurement available for exam five, and ages were matched accordingly.

Cardiovascular Health Study (CHS) recruited participants 65 years of age or older were recruited from four centres in the US, in a study designed to research cardiovascular diseases and onset of coronary heart disease. Recruiting ran from 1989 to 1999, with 5201 participants in the initial recruitment phase.

Framingham Heart Study (FHS) recruited 5209 subjects in 1948 from the town of Framingham, Massachusetts. This was the Original Cohort, further recruitment of the their offspring and grandchildren (Generation 3) was started in 1971 and 2002 respectively. Annual examinations were carried out on each of the cohorts. Subjects from Generation 3 were used in this research. ​

A total of 13,377 individuals of self-reported European ancestry were included in these four populations, with individuals taking urate-lowering medication or having self-reported gout being excluded. There was a total of 4,488 CKD cases (eGFR<60 mL/min/1.73m^2^). FHS was excluded for analyses of CKD, as there were too few CKD cases (N<10) for analysis. The cohorts were accessed through the Database of Genotype and Phenotype ([www.ncbi.nlm.nih.gov/gap](http://www.ncbi.nlm.nih.gov/gap)). The research procedures were in accordance with the ethical standards of the institutional review boards relevant to the four studies.
